# Nutritional interventions for heart failure patients who are malnourished or at risk of malnutrition or cachexia: a systematic review and meta-analysis

**DOI:** 10.1007/s10741-020-09937-9

**Published:** 2020-03-02

**Authors:** Dina Habaybeh, Mariana Bordinhon de Moraes, Adrian Slee, Christina Avgerinou

**Affiliations:** 1 Division of Medicine, University College London, London, UK; 2 Department of Public Health, Botucatu Medical School, São Paulo State University (UNESP), Sao Paulo, Brazil; 3 Department of Primary Care and Population Health, University College London, Rowland Hill Street, London, NW3 2PF UK

**Keywords:** Heart failure, Malnutrition, Nutritional interventions, Oral nutritional supplements

## Abstract

Malnutrition is common in heart failure (HF), and it is associated with higher hospital readmission and mortality rates. This review aims to answer the question whether nutritional interventions aiming to increase protein and energy intake are effective at improving outcomes for patients with HF who are malnourished or at risk of malnutrition or cachexia. Systematic searches of four databases (Medline, Embase, CINAHL and Cochrane Central Register of Controlled Trials (CENTRAL)) were conducted on 21 June 2019. Randomized controlled trials (RCTs) or other interventional studies using protein or energy supplementation for adult HF patients who are malnourished or at risk of malnutrition or cachexia were included. Two independent reviewers assessed study eligibility and risk of bias. Five studies (four RCTs and one pilot RCT) met the inclusion criteria. The majority of studies were small and of limited quality. The pooled weighted mean difference (WMD) for body weight showed a benefit from the nutritional intervention by 3.83 kg (95% confidence interval (CI) 0.17 to 7.50, *P* = 0.04) from three trials with no significant benefit for triceps skinfold thickness (WMD = − 2.14 mm, 95% CI − 9.07 to 4.79, *P* = 0.55) from two trials. The combination of personalized nutrition intervention with conventional treatment led to a decrease in all-cause mortality and hospital readmission in one study. Findings of this review suggest that nutritional interventions could potentially improve outcomes in HF patients who are malnourished or at risk of malnutrition. However, the strength of the evidence is poor, and more robust studies with a larger number of participants are needed.

**Electronic supplementary material:**

The online version of this article (10.1007/s10741-020-09937-9) contains supplementary material, which is available to authorized users.

## Introduction

Heart failure (HF) is considered as a present-day epidemic, with 26 million cases worldwide [1] causing a burden on health care systems; for example, it has been valued that 2% of the National Health Service (NHS) budget is spent on HF alone [2]. The incidence of HF tends to increase with age due to age-associated changes in the heart’s function and structure [3], making HF one of the most common reasons for hospitalization in older adults [4]. The 1-year mortality rate among HF patients admitted to hospital has been estimated at 29.6% by the recent Annual National HF Audit in England [5]. Additionally, patients with HF tend to also have a high hospital readmission rate with almost 25% of patients being readmitted within 30 days [6]. Although maintaining a good quality of life is important for patients’ survival and outlook [7], it has been shown that the quality of life for patients with HF is lower than in any other chronic disease [8].

Malnutrition is common among patients with HF [9], and it predicts worse mortality and hospital readmission outcomes [10, 11]. The prevalence of malnutrition among such a group of patients has been reported to be as high as 69% depending on the screening tool being used [12], and it can be attributed to illness-related factors, such as reduced calorie intake due to medication induced anorexia (e.g. diuretics), anxiety and the lack of energy to prepare food [13, 14]. Moreover, around 5–15% of HF patients tend to suffer from cardiac cachexia [15], defined as ‘involuntary progressive weight loss due to the reduction in skeletal muscle mass with or without depletion of adipose tissue’ [16]. Cachexia is caused by immunological and hormonal abnormalities, switching the body from an anabolic to a catabolic state by a decrease in the activity and levels of anabolic mediators such as insulin and growth hormone and an increase in activity and levels of catabolic mediators such as pro-inflammatory cytokines and glucocorticoids [17]. The above changes lead to a hypermetabolic state [18] and an increase in protein degradation [19], and therefore, result in muscle wasting.

Considering the pathophysiology of malnutrition and cachexia in HF, it has been hypothesised that the supplementation of protein or the increase in energy intake could reduce catabolic effects and increase in lean body mass tissue in these patients [20, 21]. However, no nutritional guidelines for the management of HF currently exist. Although systematic reviews have investigated the effectiveness of restrictive diets (e.g. low sodium and fluid restriction) for HF patients, no systematic review so far has focused on nutritional interventions tackling malnutrition in HF patients. Therefore, this systematic review, being the first of its kind, will focus on answering the question whether nutritional interventions aiming to increase protein or energy intake for malnourished or at risk of malnutrition or cachexia HF patients are effective at improving clinical outcomes including nutritional status, mortality and hospital readmission. The aim is to present the evidence regarding the effectiveness of nutritional interventions, which can potentially help form guidelines for nutritional support in HF patients.

## Methods

### Searches

A search strategy was created and applied with the assistance of a professional librarian to combine the following key concepts: HF, malnutrition/cachexia and oral nutrition supplements (ONS). The search strategy was applied to four databases: Embase, Medline, CINAHL and Cochrane Controlled Register of Trials (CENTRAL) on 21 June 2019. Detailed search strategy for Medline and Embase can be found in Appendix 1. Reference list searching from selected included papers was also undertaken. No restrictions were applied on language or publication date when applying the searches. A filter was applied for the Embase search to exclude conference abstracts. Findings were reported following the Preferred Reporting Items for Systematic Reviews and Meta-Analyses (PRISMA) guidelines [22]. A PRISMA checklist is provided in Appendix 2.

### Study selection

Independent screening of title, abstract and full text was performed by two reviewers (DH, MBM) who selected studies that met the inclusion criteria. Discussions took place between the two reviewers, and disagreements were resolved by discussion with a third reviewer (CA) when required. Studies that were not possible to be obtained in a full-text English version were excluded.

### Types of studies included

#### Design

Randomized controlled trials (RCTs) or other interventional studies in humans.

#### Participants

*Inclusion criteria*:

- Adult patients with a diagnosis of HF (any age > 18)
- Malnutrition, cachexia or risk of malnutrition

*Exclusion criteria*:

- Children/adolescents
- Participants without a diagnosis of HF

#### Interventions

*Inclusion criteria*: (1) ONS, (2) food enrichment, (3) any other form of protein supplementation, (4) nutrition education targeting the increase of protein or energy intake, (5) combination of any of the methods mentioned above (no time limit for the duration of intervention or time limit for follow-up was set).

*Exclusion criteria*: (1) interventions aiming to reduce salt and water intake, (2) other interventions not focussing on increasing protein or energy intake (e.g. interventions aiming to reduce cholesterol), (3) vitamin supplementation (e.g. vitamin D) unless this formed part of a wider intervention aiming to increase protein/energy.

#### Comparator

*Inclusion criteria*: studies that compared the intervention group with:

1. Standard care
2. Placebo
3. Other non-nutritional interventions applied to both the control and the nutritional intervention group, e.g. physical activity/exercise
4. Pre-intervention measurements

*Exclusion criteria*: no suitable comparator (when it was not possible to separate the effect attributed purely to a nutritional intervention).

#### Outcomes

*Inclusion criteria*:

Studies with at least one of the following primary outcomes:

1. Nutritional status assessed by any of the following:

- Anthropometry (BMI, weight, mid upper arm circumference, calf circumference, triceps skinfold thickness, lean body weight, etc.)
- Nutritional risk (measured by Mini Nutritional Assessment (MNA), Malnutrition Universal Screening Tool (MUST) or other validated tools)
- Nutritional-related outcomes that are subjectively assessed (self-reported protein and energy intake, dietary recall, changes in dietary behaviour or knowledge)

2. Hospital admission/readmission
3. Mortality

Secondary outcomes:

1. Quality of life
2. Depression
3. Physical performance
4. Functioning
5. Outcomes related to HF (e.g. breathlessness, exercise tolerance)
6. Outcomes related to malnutrition (e.g. infections, pressure sores, etc.)
7. Any other relevant clinical outcome (excluding biochemical outcomes that are unrelated to malnutrition)

*Exclusion criteria*: studies that measured exercise capacity only without measuring any of the main primary outcomes (nutrition, hospital readmission and mortality) were excluded.

### Data extraction

Data was extracted by the main author (DH), who tabulated data for each study, including author, year, country, setting, study population, sample size, number of participants in intervention and control, mean age (intervention/control), sex (female/male), type of intervention, duration of intervention, control (type of comparator), period of follow-up, number of participants completing the follow-up, outcomes assessed and main findings. The authors of the articles included in this review were contacted by e-mail when information was unclear/missing.

### Study quality assessment

The Cochrane Risk of Bias Tool for randomized controlled trials [23] was used to assess the methodological quality of the included studies. Two reviewers (DH and CA) independently assessed risk of bias.

### Data synthesis and presentation

Data from included studies was extracted and included in tables summarising the results. A random effects pooled analysis was performed for outcomes where the length of the intervention and type of intervention was comparable, and the combined post-intervention effect was calculated. Results were converted into mean ± standard deviation when possible, and the authors were contacted when unpublished data was required for the meta-analysis. Heterogeneity was calculated using I^2^ statistical test and data in the pooled analysis was presented as difference in mean and 95% confidence intervals (CIs). *P* < 0.05 was considered statistically significant. Review Manager 5 (RevMan 5) software was used. Other outcomes were presented in a narrative way.

### Study registration

The protocol of this review has been registered in the PROSPERO database (International Prospective Register of Systematic Reviews, University of York) (CRD42019142323) [24].

## Results

### Selection process

**Fig. 1:**
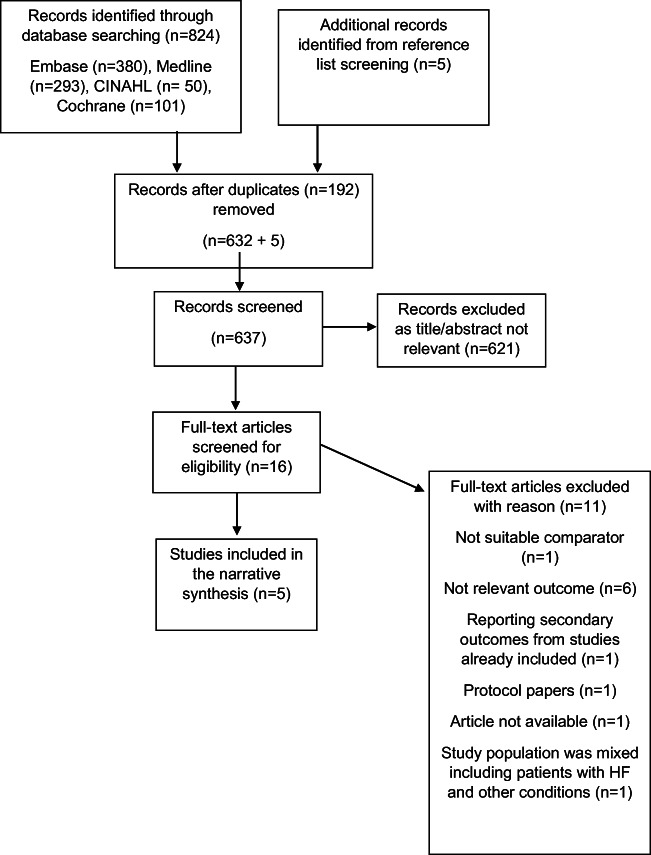
PRISMA flow diagram of selecting eligible studies

A total of 824 titles and abstracts were identified in the searches and five additional articles were identified through reference list screening of the included articles. After removing duplicates (*n* = 192), 632 + 5 articles were screened in which 621 titles and abstracts were excluded as found to be irrelevant. The remainder 16 articles were selected to be screened for eligibility. Of those 16 articles screened, 11 were rejected, as one did not have a suitable comparator [25], six did not have a relevant outcome [26–31], one was a protocol paper [32], one was reporting secondary outcomes of studies already selected to be included in the review [33], and one was rejected as the full-text English version was not available [34]. One further paper was found to be relevant, but it was excluded because the study population was mixed including patients with heart failure, acute myocardial infarction, pneumonia and chronic obstructive pulmonary disease (COPD), and the results were presented for the whole patient population without subgroup analysis for HF only [35]. Finally, five studies were included in this review. The PRISMA flow chart in Fig. 1 shows a summary of the selection process.

### Study characteristics

Among the five studies that were included in this review, four were RCTs and one was a pilot RCT. The total number of participants included in these studies was 275. The country of origin varied, including Poland [36], Sweden [37], Mexico [38], Italy [39] and Spain [40]. Table 1 shows the characteristics of the studies included in this review.

**Table 1:** Description of the studies that are included in this review

Author, year Country Setting	Study design	Study population	Sample size (*N*) Mean age (years) Sex (F/M)	Intervention Duration of intervention	Control	Follow-up period Number of participants completed follow-up	Outcomes assessed	Main findings
Aquilani et al., 2008 [39] Italy Outpatient clinic	RCT	Stable outpatient CHF patients with a normal BMI (20 < BMI < 25 kg/m^2^) and a high depletion of skeletal muscle mass (arm muscle area < 10th percentile of normal values for age and sex) with a stable daily energy protein intake over the past year providing energy ≥ 30 kcal/kg and protein > 1.1 g/kg that engage adequate daily physical activity Aged > 69	*N* = 38 (I:21, C:17) (I:73.1, C:74.5) (11/27)	Oral supplementation of EAA 8 g/day 2 months	No supplementation	2 months (I:21, C:17)	1. Anthropometric measurements (body weight, BMI, triceps skin fold thickness and arm muscle mass area) 2. Diet measurements (7-day food diary) 3. Functional measurements (power output (Watt), peak VO2, RER and 6-min walk test) (Continuous data presented as mean ± standard deviation)	1. (i) Body weight increased by > 1 kg in 80% of EAA supplemented patients (mean 2.96 kg) and in 30% of controls (mean 2.3 kg) (*P* < 0.05) (ii) A significant increase in body weight was observed in patients receiving the intervention at 2 months compared to baseline (intervention at 2 months 58.2 kg ± 7.2 vs. baseline 55.9 kg ± 17) (*P* < 0.01) (iii) No significant difference between the intervention and the control group for anthropometric measurements 2. (i) No significant difference between the intervention and the control group for dietary measurements 3. (i) A significant increase in power output was found in the IG compared with the CG (I 95 ± 25 W vs. 88 ± 22 W) (*P* < 0.01) (ii) A significant increase in peak VO2 was found in the IG compared with the CG (I 14.9 ± 1.9 ml/O_2_/kg/min vs. C 13 ± 3.5 ml/O_2_/kg/min) (*P* < 0.05) (iii) A significant increase in 6-min walk test was found in the IG compared with the CG (I 405 ± 130 m vs. 310 ± 155 m) (*P* < 0.01) (iv) No significant difference between the intervention and the control group for other functional measurements
Rozentryt et al., 2010 [36] Poland Outpatient clinic	Pilot RCT	Stable HF NYHA functional class II-IV with left ventricular ejection fraction ≤30% with 7.5% weight loss over 6 months between the ages 18–80 years	*N* = 29 (I:23, C:6) (I:52, C:49) (7/22)	High-calorie, high-protein ONS (600 kcal, 20 g protein, 72 g carbohydrates, 26 g fat) 6 weeks	Pre-intervention	6 weeks (I:20, C:5) 18 weeks (I:19, C:5)	1. Anthropometric measurements (body weight) 2. Body composition (DEXA) 3. Malnutrition-related biological parameters (albumin) 4. Quality of life (MLHFQ) 5. Left ventricular ejection fraction (echocardiogram) 6. Functional capacity (6-min walk test and peak VO2) (Continuous data presented as mean ± standard error of the mean or percent of patients)	1. (i) A significant increase in body weight in patients receiving the intervention at 6 weeks with an average weight gain of 2.0 ± 1.7 kg (3.1 ± 2.4%) (*P* = 0.0001) (ii) A significant increase in body weight in patients receiving the intervention at 18 weeks with an average weight gain of 2.3 ± 3.1 kg (3.6 ± 4.7%) (*P* = 0.007) 2. (i) A significant increase in fat tissue mass in patients receiving the intervention at 6 weeks compared to baseline with an average fat gain of 1.5 ± 1.7 kg (9.7 ± 12.7%) (*P* = 0.003) (ii) A significant increase in fat tissue mass in patients receiving the intervention at 18 weeks compared to baseline with an average fat gain of 1.6 ± 2.7 kg (10 ± 18.2%) (*P* = 0.008*)* (iii) A significant increase in lean tissue mass in patients receiving the intervention at 6 weeks compared to baseline (intervention at 6 weeks 45.49 ± 1.89 kg vs. baseline 44.97 ± 1.86 kg) (*P* = 0.019) 3. (i) No significant difference in malnutrition-related biological parameters between the intervention at 6 weeks vs. baseline and intervention at 18 weeks vs. baseline 4. (i) A significant improvement in quality of life in patients receiving the intervention at 6 weeks compared to baseline (intervention at 6 weeks MLHFQ score 37 ± 6 vs. baseline score 47 ± 5) (*P* = 0.0001) (ii) A significant improvement in quality of life in patients receiving the intervention at 18 weeks compared to baseline (intervention at 18 weeks: MLHFQ score 42 ± 7 vs. baseline score 47 ± 5) (*P* = 0.006) 5. (i) No significant difference in left ventricular ejection fraction between the intervention at 6 weeks vs. baseline and intervention at 18 weeks vs. baseline 6. (i) A significant increase in the 6 min walk test in patients receiving the intervention at 6 weeks compared to baseline (intervention at 6 weeks 410 ± 24 m vs. baseline 366 ± 23 m) (*P* = 0.02) (ii) No significant difference in other functional capacity measurements between the intervention at 6 weeks vs. baseline and intervention at 18 weeks vs. baseline
Broqvist et al., 1994 [37] Sweden Department of cardiology, University hospital (setting unstated)	RCT	Severe CHF patients NYHA functional class III-IV between the age 60–87 years	*N* = 22 (I:9, C:13) (I:70, C:73) (3/19)	500 ml daily dietary supplement (containing 30 g of protein, 30 g of fat and 87.5 g of carbohydrate and a total energy of 750 kcal) (Biosorb 1500, Pharmacia, Germany) 8 weeks	500 ml of a 1:10 diluted placebo version of the supplement	8 weeks (I:7, C:12)	1. Anthropometric measurements (weight, weight index, triceps skinfold thickness, arm muscle circumference and mid-arm circumference) 2. Muscle content of ATP, phosphocreatine, creatine, total creatine, lactate, glycogen and water (muscle biopsy analysis) 3. Diet measurements (diet history questionnaire) 4. Malnutrition-related biological parameters (albumin and transthyretin) 5. Exercise related outcomes (heart rate, blood pressure,VO2, VCO2, VE and RER) 6. Clinical HF related measurements (NYHA functional class, P-norepinephrine, P-ANP, urinary aldosterone, HF medications: digoxin, furosemide, metolazone, potassium-sparing diuretics, captopril, enalapril and nitrates) (Continuous data presented as mean ± standard error of the mean)	1. (i) A significant increase for triceps skinfold thickness was found in the IG compared with the CG (I 15.2 ± 2.3 mm vs. C 9.2 ± 0.8 mm) (*P* < 0.05) (ii) No significant difference between the intervention and the control group for the other anthropometric measurements 2. (i) No significant difference between the intervention and the control group for muscle content measurements 3. (i) A significant increase in fat intake was found in the IG compared with the CG (I 104 ± 10 g vs. C:72 ± 6 g) (*P* < 0.05*)* (ii) A significant increase in non-protein energy intake was found in the IG compared with the CG (I 2420 ± 250 kcal vs. C 1908 ± 156 kcal) (*P* < 0.05) (iii) No significant difference between the intervention and control group for other dietary measurements 4. (i) No significant difference between the intervention and control group for malnutrition-related biological parameters 5. (i) No significant difference between the intervention and the control group for exercise related outcomes 6. (i) A significant increase in P-norepinephrine in the IG compared with the CG (I 4.2 ± 0.5 nmol 1^−1^ vs. C 2.8 ± 0.4 nmol 1^−1^) (*P* < 0.05) (ii) No significant difference between the intervention and control group for other clinical HF-related measurements
Pineda-Juárez et al., 2016 [38] Mexico Outpatient clinic	RCT	Stable HF patients from a HF clinic “Instituto Nacional de Ciencias Medicas y Nutricion Salvador Zubrian” (INCMNSZ) in Mexico City aged > 18	*N* = 66 (I:34, C:32) (I:74.5, C:71) (27/39)	Resistance exercise program plus 10 g/day of Amino 2000 BCAA supplementation (5 g after breakfast and 5 g before resistance exercise) 12 weeks	Resistance exercise	12 weeks (I:29, C:26)	1.Anthropometric measurements: (body weight, height, arm, waist and hip circumference, and muscle strength) 2.Body composition (BIA) 3. Diet measurements (24-h diet recall) 4. Malnutrition-related biological parameters (albumin) 5. Stress test (METS, VO2 max, resting heart rate, exercise heart rate, resting systolic blood pressure, resting diastolic blood pressure, exercise systolic blood pressure, exercise diastolic blood pressure and treadmill time) 6. Clinical changes (intolerance decubitus, dyspnoea, oedema and fatigue) (Data presented as percentage change)	1. (i) A significant decrease in hip circumference in the IG compared with the CG (percentage change in I: − 3.1% cm vs. percentage change in C: − 1.5% cm) (*P* = 0.02) (ii) No significant difference between the IG and CG in other anthropometric measurements 2. (i) No significant difference between the intervention and control group in any body composition related variables 3. (i) No significant difference between the intervention and the control group for dietary measurements 4. (i) No significant difference between the intervention and the control group for malnutrition-related biological parameters 5. (i) A significant increase in exercise diastolic blood pressure in the IG compared with the CG (percentage change in I: + 15.4% mmHg vs. C: − 12.0% mmHg) (*P* = 0.0001) (ii) No significant difference between the intervention and control group in other stress test measurements 6. (i) A significant decrease in dyspnoea in the IG compared with the CG (*P = 0.03*) (ii) No significant difference between the intervention and control group in other clinical changes
Bonilla-Palomas et al., 2016 [40] Spain Hospital	RCT	Hospitalized HF patients diagnosed with either decompensated CHF or new onset HF who are malnourished	*N* = 120 (I:59, C:61) (I:78.6, C:79.8) (75/45)	Conventional treatment + nutritional intervention (diet optimization, specific nutritional recommendations and nutritional supplement prescriptions when nutritional goals were not reached) 6 months	Conventional treatment	12 months (I:59, C:61)	1. Composite outcome of all-case death or readmission for worsening of HF 2.All-cause death 3.Hospital readmission due to worsening of HF (Data presented as percentage and hazard ratio with 95% Confidence interval)	1. (i) A significant decrease in the composite end point of all-case death or readmission for worsening of HF in IG compared with CG (I 27.1% vs. C 60.7%) (HR 0.45; 95% CI, 0.019–0.62) (*P* = 0.0004) 2. (i) A significant decrease in all-cause death in IG compared with CG (I 20.3% vs. C 47.5%) (HR 0.37; 95% CI, 0.09–0.52) (*P* = 0.001) 3. (i) A significant decrease in hospital readmission in IG compared with CG (I 10.2% vs. C 36.1%) (HR 0.21; 95% CI, 0.09–0.52) (*P* = 0.001)

*ATP* adenosine triphosphate, *BCAA* branched chain amino acid, *BIA* bioelectrical impedance analysis, *BMI* body mass index, *C* control, *CG* control group, *CHF* chronic heart failure, *CI* confidence interval, *DEXA* dual X-ray absorptiometry, *EAA* essential amino acid, *HF* heart failure, *HR* hazard ratio, *I* intervention, *IG* intervention group, *METS* metabolic equivalents, *MLHFQ* Minnesota living with heart failure questionnaire, *NYHA* New York Heart Association, *ONS* oral nutritional supplement, *RER* respiratory exchange ratio, *peak VO2* oxygen peak, *VE* minute ventilation, *VCO2* carbon dioxide elimination, *VO2 max* maximum oxygen consumption

### Participants

More male than female participants were recruited in the selected trials (total males count 152 vs. total females count 123). Most of the studies included participants who were older than 60 (*n* = 4), while only one study recruited participants who are 42 years and older [36]. The studies selected for this review recruited HF patients who were malnourished or at risk of malnutrition or cachexia; however, the classification of HF as well as the definition and the diagnosis of malnutrition varied among the studies. One study [40] recruited participants with acute heart failure (AHF) (either decompensated chronic heart failure (CHF) or a new onset of HF) who were malnourished identified by MNA (Mini Nutritional Assessment) [41]. In another study, patients had stable CHF New York Heart Association NYHA-II (71%) and NYHA-III (29%) with severe depletion of lean muscle mass identified through having an arm muscle circumference measurement < 10th percentile of normal values for age and sex [39]. Another study recruited stable CHF NYHA-II patients who were cachectic, identified as having a weight loss of 7.5% over the past 6 months [36]. Moreover, another study included participants with stable CHF NYHA-I (60.9%), NYHA-II (31.1%), NYHA-III (6%) and NYHA-IV (2%), in which 62.1% of the participants were diagnosed as cachectic, identified by Bioelectrical Impedance Analysis (BIA) [38]. Finally, in another study patients with severe CHF (NYHA-III-IV) were recruited, the majority of whom had markedly low exercise capacity and oxygen uptake, although only two participants were diagnosed as malnourished (identified as having two nutritional-related variables as subnormal, one of which was anthropometric (weight index, triceps skinfold thickness and arm muscle circumference) and one of which was biochemical (transthyretin and albumin)). Seven patients had low serum albumin, five had low serum transthyretin and four had anthropometric values below the range. In the same study, muscle biopsies of patients with HF compared to healthy controls showed significant decreases of adenosine triphosphate (ATP), creatine, total creatine and glycogen at baseline, considered to be markers of cardiac cachexia [37].

### Interventions

There was a variety of nutritional interventions, which included ONS only in three of the studies [36, 37, 39], personalised dietary intervention with ONS prescriptions in cases where nutritional goals were not reached in one study [40] and a combination of an ONS with a resistance exercise (RE) programme in one study [38].

More specifically, the intervention consisted of:

- A high-calorie, high-protein ONS containing 20 g of protein, 26 g of fat, 72 g of carbohydrates and a total energy of 600 kcal [36]
- A 500 ml daily dietary supplement containing 30 g of protein, 30 g of fat, 87.5 g of carbohydrate and a total energy of 750 kcal [37]
- Supplementation of 8 g/day of an oral essential amino acid [39]
- Conventional treatment plus a nutritional intervention which included diet optimization, specific nutritional recommendations and nutritional supplement prescriptions when nutritional goals were not reached [40]
- A resistance exercise programme plus 10 g/day of branched chain amino acid (BCAA) supplementation [38]

### Intervention length, adherence to intervention and follow-up period

There was variation in the length of interventions for the studies included in this review with the longest duration of intervention being 6 months [40] and the shortest 6 weeks [36]. Other studies included had a duration of intervention of 8 weeks [37], 12 weeks [38] and finally 2 months [39]. The adherence to the intervention was not reported for most of the studies (*n* = 4); only one study reported adherence which was measured through counting the number of amino acid packs remaining after the end of the intervention period (2 months), and judgement of patient’s reliability was made through taking venous blood samples and measuring fasting plasma leucine concentration. Although the compliance rate was not reported, the authors assumed that the compliance was satisfactory [39]. The follow-up period for the selected studies ranged from 6 weeks [36] to 12 months [40].

### Risk of bias

**Fig. 2:**
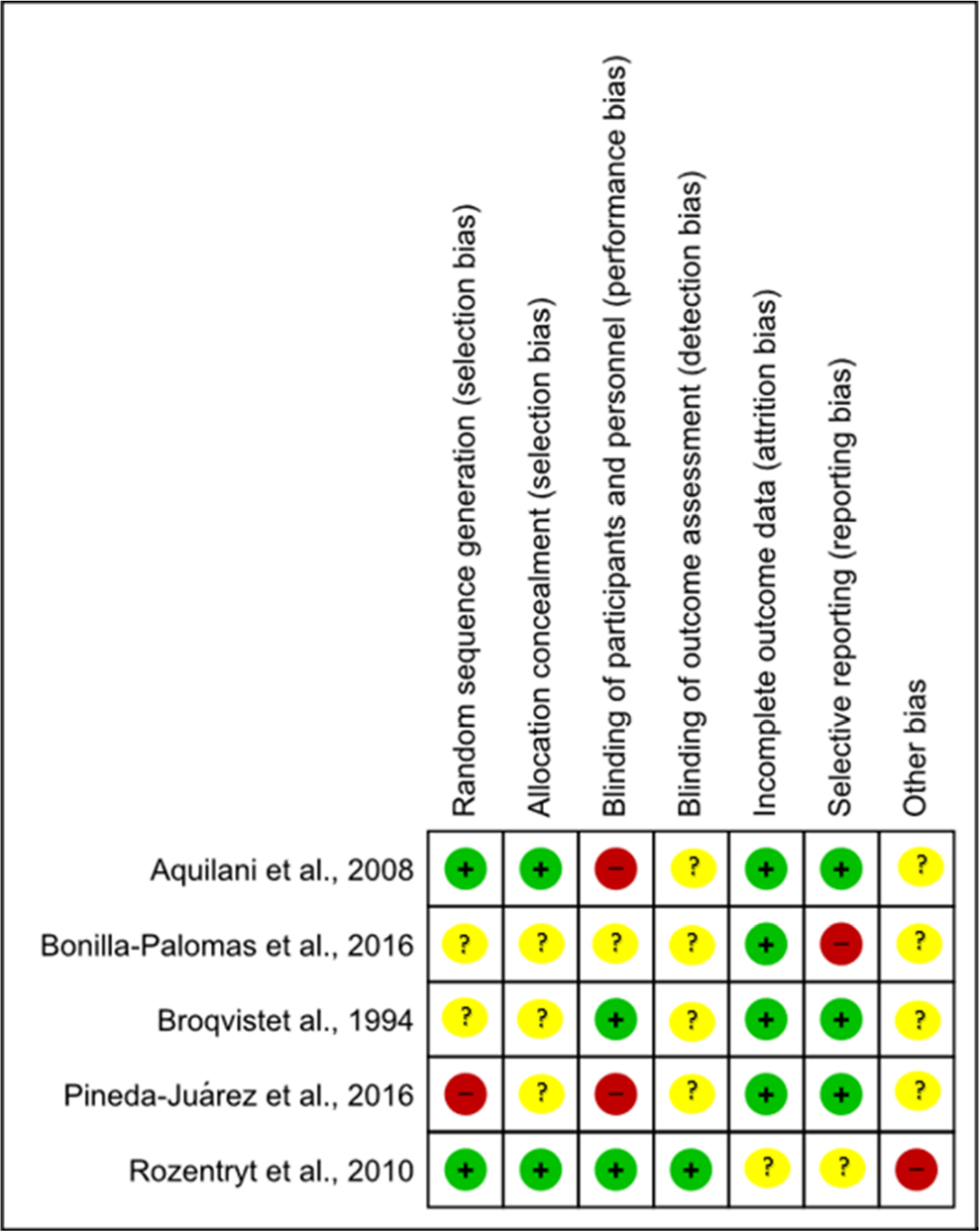
Risk of bias assessment using the Cochrane Risk of Bias Tool for RCTs. KEY: (+)/Green = Low risk (−)/Red = High risk (?)/Yellow = Unclear.

The risk of bias assessment for the studies included in this review is presented in Fig. 2. Random sequence generation had a high risk in one of the studies with other studies either being low (*n* = 2) or unclear (*n* = 2). Allocation concealment (selection bias) was either low risk (*n* = 2) or unclear (*n* = 3) in the studies included. Blinding of participants and personnel (performance bias) was high risk in two studies, unclear in one study and the remainder (*n* = 2) low risk. Blinding of outcome assessment (detection bias) was unclear in most of the studies (*n* = 4) with one of the studies having low risk. Most of the studies (*n* = 4) had low risk for incomplete outcome data (attrition bias) with only one of the studies being unclear. Selection reporting risk of bias was high in one of the studies, low for three studies and unclear for one study. Other risk of bias was high in one of the studies with the rest of the studies being unclear (*n* = 4).

### Primary outcomes

#### Nutritional status

##### Anthropometry and body composition

Of the five studies included, four studies assessed anthropometry, and two studies assessed body composition, with mixed findings across the studies. In one study, the supplementation of 8 g of essential amino acids (EAA) per day for 2 months did not lead to significant changes in anthropometric measurements when comparing the intervention group with the control group receiving no supplementation. However, a significant increase in body weight for the EAA supplemented group was observed compared to their baseline body weight [39]. Another study showed that the supplementation of 500 ml daily dietary supplement for 8 weeks significantly increased triceps skinfold thickness during the 8-week follow-up period in the supplemented intervention group when compared to the control group receiving a placebo [37]. Significant benefits were also observed in another study by Rozentryt et at. [36], which found that the supplementation of a high-caloric, high-protein ONS for 6 weeks increased body weight and fat tissue mass measured by dual X-ray absorptiometry (DEXA) during the 6-week and 18-week follow-up period, and a significant increase in lean tissue mass measured by DEXA was observed during the 6-week follow-up period only. Nonetheless, this study was a pilot RCT and data was analysed in the form of pre-/post-intervention comparison at 6 weeks and 12 weeks for the intervention arm only, with no comparison data available between ONS and placebo.

In another study, the combined intervention of resistance exercise (RE) program with the daily nutritional supplementation of 10 g of BCAA for 12 weeks resulted in a significant reduction in hip circumference during the 12-week follow-up period when compared with the control group receiving resistance exercise program only. A reduction in waist circumference and an increase in muscle strength were observed in both groups (RE and RE + ONS) at the end of the programme, but changes were not significant between the groups. Moreover, body composition parameters assessed by BIA did not significantly improve with the addition of ONS to the exercise programme. The authors assumed that fat mass could have decreased, and muscle mass increased as a result of the anabolic effect of exercise. They also hypothesised that reduced hip and waist circumference might be related to reduced third space water retention in both groups, probably associated with increased albumin concentration [38].

##### Body weight

**Fig. 3:**
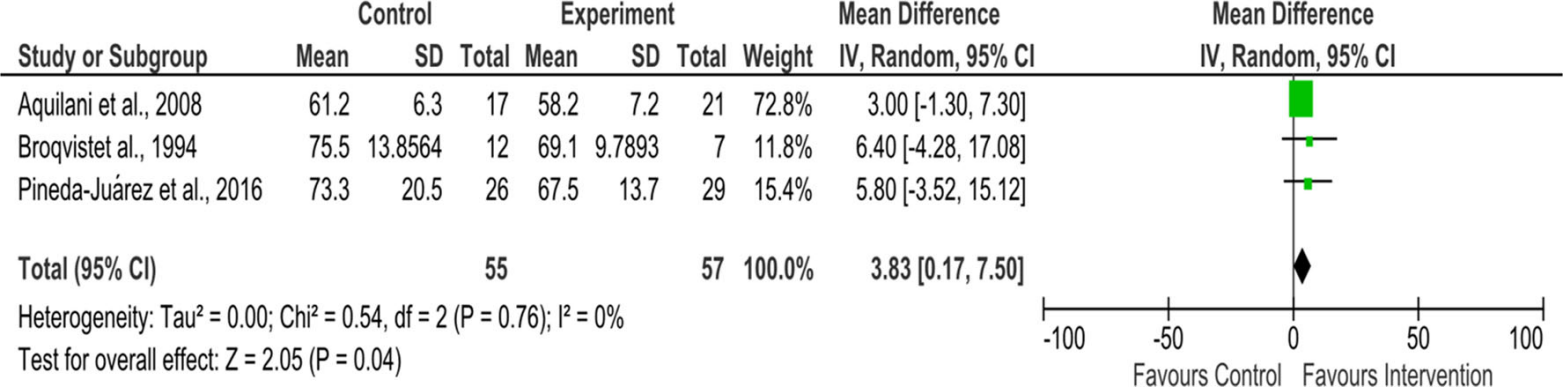
Forest plot for body weight (kg) (nutritional intervention vs. no nutritional intervention)

Three studies that assessed body weight were eligible to be included in the meta-analysis. The intervention length was 8 weeks [37], 12 weeks [38] and 2 months [39]. No significant heterogeneity was detected (I^2^ = 0%, *P* = 0.76), and pooled results have shown that the nutritional intervention significantly increased body weight in HF patients (112 participants; weighed mean difference = 3.83 kg, 95% CI 0.17 to 7.50, *P* = 0.04) (Fig. 3).

##### Triceps skinfold thickness

**Fig. 4:**
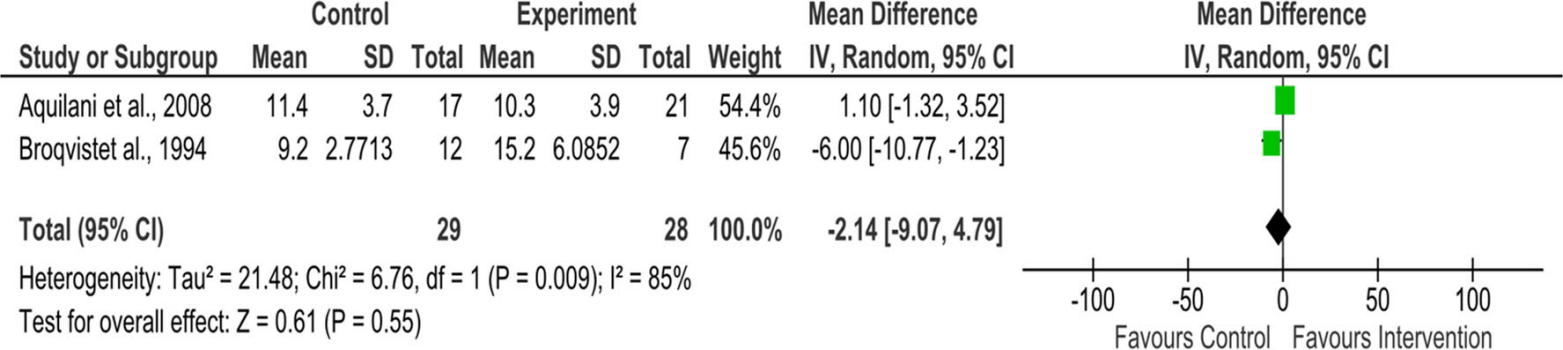
Forest plot for triceps skinfold thickness (mm) (nutritional intervention vs. no nutritional intervention)

Two studies that assessed triceps skinfold thickness were eligible to be included in the meta-analysis. The intervention length was 8 weeks [37] and 2 months [39]. Significant heterogeneity was detected (I^2^ = 85%, *P* = 0.009), and pooled results have shown that the nutritional intervention had no effect on triceps skinfold thickness for HF patients (57 participants; weighted mean difference = − 2.14 mm, 95% CI − 9.07 to 4.79, *P* = 0.55) (Fig. 4).

##### Dietary assessment

Three studies assessed dietary intake. Two of these studies showed that the nutritional intervention did not result in any significant improvement in nutritional intake measured by 7-day food recall method [39] and 24-h dietary recall method [38]. However, significant improvement in nutritional intake measured by the modified dietary history method was observed in one of the studies, in which ONS supplementation resulted in an increase in fat and non-protein energy intake during the 8-week follow-up period when compared with the control group receiving a placebo [37].

##### Malnutrition-related biological parameters

None of the three included studies [36–38] that assessed malnutrition-related biological parameters showed any significant outcomes.

#### Hospital readmission and mortality

Only one of the studies reported hospital readmission and mortality. In this study, the intervention consisted of a 6-week conventional treatment with diet optimisation, specific nutritional recommendations and nutritional supplement prescriptions when nutritional goals were not reached. This resulted in a significant reduction in the composite end point (death from all causes or readmission for worsening of HF), as well as isolated all-cause mortality and hospital readmission rates during the 12-month follow-up period when compared to the control group receiving the conventional treatment alone [40].

### Secondary outcomes

#### Exercise capacity

Four included studies assessed outcomes that related to exercise capacity, with only one study showing no significant effects on giving a nutritional intervention on exercise capacity-related outcomes [37]. However, positive benefits were observed in the other studies. For instance, in the study by Aquilani et al. [39], the supplementation of 8 g/day of EAA for 2 months led to a significant increase in power output, peak VO2 (maximum rate of oxygen consumption measured during incremental exercise) and 6-min walk functional test during the 2 month follow-up period when compared with the control group receiving no supplementation. Moreover, in a study by Rozentryt et al. [36], the nutritional intervention of supplementing a high-caloric, high-protein ONS for 6 weeks resulted in a significant increase in 6-min walk functional test during the 6-week follow-up period but not for the 18-week follow-up period when compared with pre-intervention values. Furthermore, another study showed that the combined intervention of resistance exercise program with the daily nutritional supplementation of 10 g of BCAA for 2 months resulted in a significant increase in exercise diastolic blood pressure during the 2-month follow-up period when compared with the control group receiving resistance exercise program only [38].

#### Quality of life

Quality of life was assessed in one study only, which has found that the nutritional intervention consisting of supplementing a high-caloric, high-protein ONS for 6 weeks resulted in a significant increase in quality of life during the 6-week and 18-week follow-up period when compared to pre-intervention values [36].

#### Other clinical heart failure related outcomes

Three included studies assessed clinical outcomes that relate to HF. The effect of the nutritional intervention and the outcomes being assessed among the studies varied; for example, in one study, the nutritional intervention of supplementing a high-caloric, high-protein ONS for 6 weeks resulted in a non-significant difference in left ventricular ejection fraction during the 6-week and 18-week follow-up period when compared to pre-intervention values [36]. However, in another study, the supplementation of a 500-ml daily dietary supplement for 8 weeks resulted in a significant increase in P-norepinephrine during the 8-week follow-up period when compared to the control group receiving a placebo [37]. Moreover, positive benefits were observed in another study in which the combined intervention of resistance exercise program with the daily nutritional supplementation of 10 g of BCAA for 2 months resulted in a significant decrease in dyspnoea when compared with the control group receiving resistance exercise program only [38].

## Discussion

### Summary of findings

Five studies were identified (four RCTs and one pilot RCT) reporting conflicting results on different outcomes being tested. Pooled analysis of data from three studies [37–39] showed a significant increase in body weight with ONS supplementation. This is an interesting finding, given that there was no significant weight change in the isolated studies included in the analysis, with the exception of one study which has found that body weight significantly increased in the EAA supplemented group when compared to their baseline body weight [39], and this could be potentially explained by a lack of power in the individual studies. However, pooled analysis of data from two studies [37, 39] showed no significant benefit on triceps skinfold thickness. Moreover, one study found that the combination of conventional treatment with personalized nutritional intervention for 6 months led to a significant decrease in all-cause mortality and hospital readmission rates during the 12-month follow-up period [40].

The results for other outcomes were mixed with some benefits from nutritional intervention on outcomes relating to body composition post-intervention compared to baseline, but without comparison with control group post-intervention [36], non-protein and fat energy intake [37], exercise capacity-related outcomes [36, 39], quality of life [36] and clinical HF-related outcomes such as dyspnoea [38], with no significant effect on malnutrition-related biological parameters [36–38]. Inconsistency in results across the studies could potentially be explained by the differences in the severity of HF, as well as the setting where the study was undertaken. The overall quality of the studies was low, with recruitment of a small number of participants and no formal sample size calculation in most cases.

### Comparison with existing literature

Observational studies have shown that patients with HF tend to have insufficient energy and protein intake [9, 42], and it has been suggested that nutritional interventions aiming to increase protein or energy could lead to a better coping mechanism in the anabolic/catabolic imbalances caused by the inflammation process and neurohormone activation, which are common among HF patients [43]. Although some researchers have argued that nutritional interventions aiming to increase protein or calorie intake do not reverse the physiological consequences involved in cardiac cachexia [44, 45], this review has demonstrated that the 6-week supplementation of a high-caloric, high-protein ONS for cachectic HF patients led to a significant increase in body composition values measured by DEXA, such as the increase in fat tissue mass during the 6-week and 18-week follow-up period, as well as an increase in lean tissue mass during the 6-week follow-up period when comparing post-intervention with baseline measurements [36]. Such findings are important, as the increase in fat mass has been linked to better survival outcomes and the increase in lean body mass has been linked to a better quality of life among HF patients [46].

Furthermore, data from a recent systematic review has shown that malnutrition among HF patients is associated with higher hospital readmission and mortality rates [47] and this can consequently lead to a series of other negative long-term outcomes. For instance, frequent hospital readmissions among HF patients have been associated with a decrease in quality of life and an increase in healthcare costs related to HF [48, 49]. Given such information, the finding that personalised nutritional intervention for malnourished HF patients led to a reduction in hospital readmission and mortality [40] is important, as the integration of nutritional support in standard care for HF patients could potentially contribute to better outcomes for such patients. However, hospital readmission and mortality were measured as outcomes in only one of the included studies, and the effectiveness of nutritional interventions would have to be tested in a larger number of participants before safe conclusions can be made.

It is also important to report here key findings of a multicentre RCT where older, malnourished, hospitalised adults suffering from various conditions including heart failure, acute myocardial infarction, pneumonia or COPD were randomised to receive a specialised ONS consisting of a high protein content and beta-hydroxy-beta methylbutyrate (HMB) (HP-HMB) (*n* = 328) or a placebo supplement (*n* = 324). Among the recruited patients, 157 had HF (HP-HMB *n* = 79, placebo *n* = 78). No effects were observed for the primary composite endpoint (event of death or readmission within 90 days post-discharge). However, the 90-day mortality rate was lower with HP-HMB compared to placebo (4.8% vs. 9.7%; relative risk 0.49, 95% confidence interval [CI], 0.27 to 0.90). Compared with placebo, HP-HMB resulted in improved odds of better nutritional status at day 90, and an increase in body weight at day 30. No between-group differences were observed for 90-day readmission rate, length of stay in hospital or activities of daily living [35].

In terms of outcomes that relate to exercise capacity, results from this review have shown that nutritional intervention consisting of high protein or energy could induce possible positive effects on functional outcomes such as 6-min walk test and VO2 max (which is a predictor of exercise performance) [36, 39]. While this was a secondary outcome in this study, other studies in the literature have also shown that the supplementation of individualised amino acids such as taurine [50] or the combination of essential and semi-essential amino acids [51] could improve exercise capacity in HF patients who are not malnourished. Though this is beyond the scope of this review, it is important to mention that the benefits of supplementing amino acids go beyond just their anabolic effects, but rather they are important for the maintenance of normal physiological functions [52], which should also be considered when understanding the physiological benefit of protein or amino acid supplementation.

### Strengths and limitations of this review

This review has several strengths, one being that it is, to this date, the first systematic review focusing on whether nutritional interventions aiming to increase protein or energy intake are effective at improving outcomes for patients with HF who are malnourished or at risk of malnutrition. Additionally, a rigorous methodology was followed, and more specifically, two independent reviewers were involved during screening, assessing the risk of bias and extracting data from the studies.

However, certain limitations of this review also exist, and these are related to methodological challenges. Importantly, the definition and the method of diagnosing malnutrition, risk of malnutrition or cachexia varied across the studies. Although there is a significant overlap between malnutrition and cachexia [53], these two conditions are not synonymous; hence, one could argue that the total population could potentially be heterogeneous, despite sharing common features. Additionally, adherence to the nutritional interventions was either insufficiently reported or not reported. Furthermore, only one study reported on hospital readmission and mortality, despite them being important outcomes when considering malnourished HF patients. Also, dietary intake measured by dietary recall methods could be subject to recall bias. Moreover, most of the studies included recruited a small number of participants with high attrition rates, and they did not have a formal sample size calculation, which means that they may have been underpowered. Finally, other limitations of this review are that no grey literature search was undertaken, and non-English articles were excluded; hence, relevant articles could have been missed.

### Implications for practice and research

Findings of this review suggest that there is some evidence that protein or energy nutritional supplements could potentially improve body weight, hospital readmission, and mortality in patients with HF who are malnourished or at risk of malnutrition. However, it is important to interpret the results from this review with caution, given that most of the evidence is generated from low- to moderate-quality studies with a small sample size and a short follow-up period. Therefore, this review calls for better-quality studies to be conducted in the future with robust methodology, adequate power size calculation, careful selection of participants and assessment of important clinical outcomes as mortality and hospital readmission. Additionally, consistent measurements and definition of malnutrition should be considered with further exploration on the type of supplement and dosage required to reach optimum benefits along with the physiological mechanisms involved.

## Conclusion

There is a potential benefit from oral nutritional supplements to increase body weight in patients with heart failure who are malnourished or at risk of malnutrition, and a potential benefit from individualized dietary intervention in reducing mortality and hospital readmission. However, the quality of the evidence is low, and no recommendations can be currently made to inform clinical practice. More robust, better-quality RCTs with a larger number of participants are needed to establish the effectiveness of nutritional interventions for malnutrition in heart failure.

## Electronic supplementary material

ESM 1 (DOCX 16 kb)

ESM 2 (DOCX 18 kb)
