# Supplementary material for: Nutritional interventions for heart failure patients who are malnourished or at risk of malnutrition or cachexia: a systematic review and meta-analysis
Source: Heart Fail Rev. 2020 Mar 2;26(5):1103–18. doi: 10.1007/s10741-020-09937-9 (PMC8310486; doi:10.1007/s10741-020-09937-9)
Supplement: Supplementary file 1 — (DOCX 16 kb) [file 10741_2020_9937_MOESM1_ESM.docx]

**Appendix 2:** Medline and Embase search strategies

Medline search strategy:

| \| 1. exp Heart Failure/ \|  \| \| --- \| --- \| \| 2. ((heart or cardiac or myocardial or ventricular) adj3 failure).mp. [mp=title, abstract, original title, name of substance word, subject heading word, floating sub-heading word, keyword heading word, organism supplementary concept word, protocol supplementary concept word, rare disease supplementary concept word, unique identifier, synonyms] \|  \| \| 3. (heart adj2 decompensation).mp. [mp=title, abstract, original title, name of substance word, subject heading word, floating sub-heading word, keyword heading word, organism supplementary concept word, protocol supplementary concept word, rare disease supplementary concept word, unique identifier, synonyms] \|  \| \| 4. (ventric* adj3 dysfunction).mp. [mp=title, abstract, original title, name of substance word, subject heading word, floating sub-heading word, keyword heading word, organism supplementary concept word, protocol supplementary concept word, rare disease supplementary concept word, unique identifier, synonyms] \|  \| \| 5. CHF.mp. [mp=title, abstract, original title, name of substance word, subject heading word, floating sub-heading word, keyword heading word, organism supplementary concept word, protocol supplementary concept word, rare disease supplementary concept word, unique identifier, synonyms] \|  \| \| 6. 1 or 2 or 3 or 4 or 5 \|  \| \| 7. malnutrition/ or exp deficiency diseases/ or exp severe acute malnutrition/ \|  \| \| 8. Wasting Syndrome/ \|  \| \| 9. Nutrition Disorders/ \|  \| \| 10. emaciation/ or cachexia/ \|  \| \| 11. maln*.mp. [mp=title, abstract, original title, name of substance word, subject heading word, floating sub-heading word, keyword heading word, organism supplementary concept word, protocol supplementary concept word, rare disease supplementary concept word, unique identifier, synonyms] \|  \| \| 12. (under nutrition or undernutrition or under nourish* or undernourish*).mp. [mp=title, abstract, original title, name of substance word, subject heading word, floating sub-heading word, keyword heading word, organism supplementary concept word, protocol supplementary concept word, rare disease supplementary concept word, unique identifier, synonyms] \|  \| \| 13. nutri* deficien*.mp. [mp=title, abstract, original title, name of substance word, subject heading word, floating sub-heading word, keyword heading word, organism supplementary concept word, protocol supplementary concept word, rare disease supplementary concept word, unique identifier, synonyms] \|  \| \| 14. nutritionally at risk.mp. [mp=title, abstract, original title, name of substance word, subject heading word, floating sub-heading word, keyword heading word, organism supplementary concept word, protocol supplementary concept word, rare disease supplementary concept word, unique identifier, synonyms] \|  \| \| 15. low bmi.mp. [mp=title, abstract, original title, name of substance word, subject heading word, floating sub-heading word, keyword heading word, organism supplementary concept word, protocol supplementary concept word, rare disease supplementary concept word, unique identifier, synonyms] \|  \| \| 16. low body mass index.mp. [mp=title, abstract, original title, name of substance word, subject heading word, floating sub-heading word, keyword heading word, organism supplementary concept word, protocol supplementary concept word, rare disease supplementary concept word, unique identifier, synonyms] \|  \| \| 17. wasting.mp. [mp=title, abstract, original title, name of substance word, subject heading word, floating sub-heading word, keyword heading word, organism supplementary concept word, protocol supplementary concept word, rare disease supplementary concept word, unique identifier, synonyms] \|  \| \| 18. (cachexia or cachectic).mp. [mp=title, abstract, original title, name of substance word, subject heading word, floating sub-heading word, keyword heading word, organism supplementary concept word, protocol supplementary concept word, rare disease supplementary concept word, unique identifier, synonyms] \|  \| \| 19. emaciat*.mp. [mp=title, abstract, original title, name of substance word, subject heading word, floating sub-heading word, keyword heading word, organism supplementary concept word, protocol supplementary concept word, rare disease supplementary concept word, unique identifier, synonyms] \|  \| \| 20. mal-nutrition.mp. [mp=title, abstract, original title, name of substance word, subject heading word, floating sub-heading word, keyword heading word, organism supplementary concept word, protocol supplementary concept word, rare disease supplementary concept word, unique identifier, synonyms] \|  \| \| 21. malnourish*.mp. [mp=title, abstract, original title, name of substance word, subject heading word, floating sub-heading word, keyword heading word, organism supplementary concept word, protocol supplementary concept word, rare disease supplementary concept word, unique identifier, synonyms] \|  \| \| 22. Thinness/ \|  \| \| 23. (under weight or underweight).mp. [mp=title, abstract, original title, name of substance word, subject heading word, floating sub-heading word, keyword heading word, organism supplementary concept word, protocol supplementary concept word, rare disease supplementary concept word, unique identifier, synonyms] \|  \| \| 24. or/7-23 \|  \| \| 25. 6 and 24 \|  \| \| 26. nutrition therapy/ or diet therapy/ \|  \| \| 27. Dietary Supplements/ \|  \| \| 28. Food, Fortified/ \|  \| \| 29. dh.fs. \|  \| \| 30. rh.fs. \|  \| \| 31. ONS.mp. [mp=title, abstract, original title, name of substance word, subject heading word, floating sub-heading word, keyword heading word, organism supplementary concept word, protocol supplementary concept word, rare disease supplementary concept word, unique identifier, synonyms] \|  \| \| 32. oral nutritional supplement*.mp. [mp=title, abstract, original title, name of substance word, subject heading word, floating sub-heading word, keyword heading word, organism supplementary concept word, protocol supplementary concept word, rare disease supplementary concept word, unique identifier, synonyms] \|  \| \| 33. sip feed*.mp. [mp=title, abstract, original title, name of substance word, subject heading word, floating sub-heading word, keyword heading word, organism supplementary concept word, protocol supplementary concept word, rare disease supplementary concept word, unique identifier, synonyms] \|  \| \| 34. ((nutrition* or diet*) adj8 (intervention* or rehabilit* or education* or advice or counselling or counseling or supplement* or therap*)).mp. [mp=title, abstract, original title, name of substance word, subject heading word, floating sub-heading word, keyword heading word, organism supplementary concept word, protocol supplementary concept word, rare disease supplementary concept word, unique identifier, synonyms] \|  \| \| 35. 26 or 27 or 28 or 29 or 30 or 31 or 32 or 33 or 34 \|  \| \| 36. 25 and 35 \|  \| |
| --- | --- | --- | --- | --- | --- | --- | --- | --- | --- | --- | --- | --- | --- | --- | --- | --- | --- | --- | --- | --- | --- | --- | --- | --- | --- | --- | --- | --- | --- | --- | --- | --- | --- | --- | --- | --- | --- | --- | --- | --- | --- | --- | --- | --- | --- | --- | --- | --- | --- | --- | --- | --- | --- | --- | --- | --- | --- | --- | --- | --- | --- | --- | --- | --- | --- | --- | --- | --- | --- | --- | --- | --- |

Embase search strategy:

| \| 1. exp Heart Failure/ \|  \| \| --- \| --- \| \| 2. ((heart or cardiac or myocardial or ventricular) adj3 failure).mp. [mp=title, abstract, original title, name of substance word, subject heading word, floating sub-heading word, keyword heading word, organism supplementary concept word, protocol supplementary concept word, rare disease supplementary concept word, unique identifier, synonyms] \|  \| \| 3. (heart adj2 decompensation).mp. [mp=title, abstract, original title, name of substance word, subject heading word, floating sub-heading word, keyword heading word, organism supplementary concept word, protocol supplementary concept word, rare disease supplementary concept word, unique identifier, synonyms] \|  \| \| 4. (ventric* adj3 dysfunction).mp. [mp=title, abstract, original title, name of substance word, subject heading word, floating sub-heading word, keyword heading word, organism supplementary concept word, protocol supplementary concept word, rare disease supplementary concept word, unique identifier, synonyms] \|  \| \| 5. CHF.mp. [mp=title, abstract, original title, name of substance word, subject heading word, floating sub-heading word, keyword heading word, organism supplementary concept word, protocol supplementary concept word, rare disease supplementary concept word, unique identifier, synonyms] \|  \| \| 6. 1 or 2 or 3 or 4 or 5 \|  \| \| 7. malnutrition/ \|  \| \| 8. Wasting Syndrome/ \|  \| \| 9. emaciation/ or cachexia/ \|  \| \| 10. maln*.mp. [mp=title, abstract, original title, name of substance word, subject heading word, floating sub-heading word, keyword heading word, organism supplementary concept word, protocol supplementary concept word, rare disease supplementary concept word, unique identifier, synonyms] \|  \| \| 11. (under nutrition or undernutrition or under nourish* or undernourish*).mp. [mp=title, abstract, original title, name of substance word, subject heading word, floating sub-heading word, keyword heading word, organism supplementary concept word, protocol supplementary concept word, rare disease supplementary concept word, unique identifier, synonyms] \|  \| \| 12. nutri* deficien*.mp. [mp=title, abstract, original title, name of substance word, subject heading word, floating sub-heading word, keyword heading word, organism supplementary concept word, protocol supplementary concept word, rare disease supplementary concept word, unique identifier, synonyms] \|  \| \| 13. nutritionally at risk.mp. [mp=title, abstract, original title, name of substance word, subject heading word, floating sub-heading word, keyword heading word, organism supplementary concept word, protocol supplementary concept word, rare disease supplementary concept word, unique identifier, synonyms] \|  \| \| 14. low bmi.mp. [mp=title, abstract, original title, name of substance word, subject heading word, floating sub-heading word, keyword heading word, organism supplementary concept word, protocol supplementary concept word, rare disease supplementary concept word, unique identifier, synonyms] \|  \| \| 15. low body mass index.mp. [mp=title, abstract, original title, name of substance word, subject heading word, floating sub-heading word, keyword heading word, organism supplementary concept word, protocol supplementary concept word, rare disease supplementary concept word, unique identifier, synonyms] \|  \| \| 16. wasting.mp. [mp=title, abstract, original title, name of substance word, subject heading word, floating sub-heading word, keyword heading word, organism supplementary concept word, protocol supplementary concept word, rare disease supplementary concept word, unique identifier, synonyms] \|  \| \| 17. (cachexia or cachectic).mp. [mp=title, abstract, original title, name of substance word, subject heading word, floating sub-heading word, keyword heading word, organism supplementary concept word, protocol supplementary concept word, rare disease supplementary concept word, unique identifier, synonyms] \|  \| \| 18. emaciat*.mp. [mp=title, abstract, original title, name of substance word, subject heading word, floating sub-heading word, keyword heading word, organism supplementary concept word, protocol supplementary concept word, rare disease supplementary concept word, unique identifier, synonyms] \|  \| \| 19. mal-nutrition.mp. [mp=title, abstract, original title, name of substance word, subject heading word, floating sub-heading word, keyword heading word, organism supplementary concept word, protocol supplementary concept word, rare disease supplementary concept word, unique identifier, synonyms] \|  \| \| 20. malnourish*.mp. [mp=title, abstract, original title, name of substance word, subject heading word, floating sub-heading word, keyword heading word, organism supplementary concept word, protocol supplementary concept word, rare disease supplementary concept word, unique identifier, synonyms] \|  \| \| 21. underweight/ \|  \| \| 22. (under weight or underweight).mp. [mp=title, abstract, original title, name of substance word, subject heading word, floating sub-heading word, keyword heading word, organism supplementary concept word, protocol supplementary concept word, rare disease supplementary concept word, unique identifier, synonyms] \|  \| \| 23. or/7-22 \|  \| \| 24. 6 and 23 \|  \| \| 25. diet therapy/ \|  \| \| 26. diet supplementation/ \|  \| \| 27. fortified food/ \|  \| \| 28. rh.fs. \|  \| \| 29. ONS.mp. [mp=title, abstract, original title, name of substance word, subject heading word, floating sub-heading word, keyword heading word, organism supplementary concept word, protocol supplementary concept word, rare disease supplementary concept word, unique identifier, synonyms] \|  \| \| 30. oral nutritional supplement*.mp. [mp=title, abstract, original title, name of substance word, subject heading word, floating sub-heading word, keyword heading word, organism supplementary concept word, protocol supplementary concept word, rare disease supplementary concept word, unique identifier, synonyms] \|  \| \| 31. sip feed*.mp. [mp=title, abstract, original title, name of substance word, subject heading word, floating sub-heading word, keyword heading word, organism supplementary concept word, protocol supplementary concept word, rare disease supplementary concept word, unique identifier, synonyms] \|  \| \| 32. ((nutrition* or diet*) adj8 (intervention* or rehabilit* or education* or advice or counselling or counseling or supplement* or therap*)).mp. [mp=title, abstract, original title, name of substance word, subject heading word, floating sub-heading word, keyword heading word, organism supplementary concept word, protocol supplementary concept word, rare disease supplementary concept word, unique identifier, synonyms] \|  \| \| 33. protein diet/ \|  \| \| 34. nutrition education/ \|  \| \| 35. 25 or 26 or 27 or 28 or 29 or 30 or 31 or 32 or 33 or 34 \|  \| \| 36. 24 and 35 \|  \| \| 37. limit 36 to conference abstract \|  \| \| 38. 36 not 37 \|  \| |
| --- | --- | --- | --- | --- | --- | --- | --- | --- | --- | --- | --- | --- | --- | --- | --- | --- | --- | --- | --- | --- | --- | --- | --- | --- | --- | --- | --- | --- | --- | --- | --- | --- | --- | --- | --- | --- | --- | --- | --- | --- | --- | --- | --- | --- | --- | --- | --- | --- | --- | --- | --- | --- | --- | --- | --- | --- | --- | --- | --- | --- | --- | --- | --- | --- | --- | --- | --- | --- | --- | --- | --- | --- | --- | --- | --- | --- |
